# Characteristics of germline DNA damage response gene mutations in ovarian cancer in Southwest China

**DOI:** 10.1038/s41598-024-52707-y

**Published:** 2024-03-20

**Authors:** Kaiyu Fu, Qingli Li, Jie Wang, Mengpei Zhang, Xinyu Yan, Kemin Li, Liang Song, Lan Zhong, Yu Ma, Jinghong Chen, Jing Zeng, Danqing Wang, Di Shao, Shida Zhu, Rutie Yin

**Affiliations:** 1 Department of Obstetrics and Gynecology, West China Second University Hospital, Sichuan University, Chengdu, Sichuan China; 2 Laboratory of Molecular Epidemiology of Birth Defects, West China Second University Hospital, Sichuan University, Chengdu, Sichuan China; 3 BGI Genomics, BGI-Shenzhen, Shenzhen, China; 4 College of Life Sciences, University of Chinese Academy of Sciences, Beijing, China

**Keywords:** Genetics, Oncology

## Abstract

DNA damage response (DDR) pathways are responsible for repairing endogenous or exogenous DNA damage to maintain the stability of the cellular genome, including homologous recombination repair (HRR) pathway, mismatch repair (MMR) pathway, etc. In ovarian cancer, current studies are focused on HRR genes, especially *BRCA1/2*, and the results show regional and population differences. To characterize germline mutations in DDR genes in ovarian cancer in Southwest China, 432 unselected ovarian cancer patients underwent multi-gene panel testing from October 2016 to October 2020. Overall, deleterious germline mutations in DDR genes were detected in 346 patients (80.1%), and in *BRCA1/2* were detected in 126 patients (29.2%). The prevalence of deleterious germline mutations in *BRCA2* is higher than in other studies (patients are mainly from Eastern China), and so is the mismatch repair genes. We identified three novel *BRCA1/2* mutations, two of which probably deleterious (*BRCA1* p.K1622* and *BRCA2* p.L2987P). Furthermore, we pointed out that deleterious mutations of *FNACD2* and *RECQL4* are potential ovarian cancer susceptibility genes and may predispose carriers to ovarian cancer. In conclusion, our study highlights the necessity of comprehensive germline mutation detection of DNA damage response genes in ovarian cancer patients, which is conducive to patient management and genetic counseling.

## Introduction

Ovarian cancer is the third most common gynecological malignancy, accounting for 3.4% of all malignancies and 4.7% cancer deaths among women worldwide in 2020^1^. In China, ovarian cancer disproportionately affects women's health, with approximately 57,090 newly diagnosed cases and 39,306 cancer-related deaths in 2022, showing an upward trend^2^. Despite a gradual decline in the incidence and mortality of ovarian cancer in the United States, the mortality rate still ranks highest among malignancies of the female reproductive system, posing a significant threat to women's health^3^. As for now, most ovarian cancer patients have local or distant dissemination at the time of diagnosis due to the lack of effective screening and early diagnosis methods. The prognosis of ovarian cancer is poor, with limited improvement in the long term. In recent years, the in-depth understanding of the genomic characteristics of ovarian cancer has brought breakthroughs to the treatment of patients, especially the development of anti-angiogenesis drugs and poly (ADP-ribose) polymerase (PARP) inhibitors.

Part of ovarian cancer is known to be heritable, accompany with inherited deleterious gene mutations. Genetic alteration in HRR genes may confer increased risk of multiple genetically-related malignancies, such as ovarian cancer, breast cancer, pancreatic cancers, especially *BRCA1* and *BRCA2*^4,5^. HRR is an error-free pathway for DNA double strands break repairing, and *BRCA1/2* are the core genes of this pathway. Thus, germline mutations in HRR genes may lead to errors accumulation and predispose to malignancies such as ovarian cancer. By the age of 70, the average cumulative risks of ovarian cancer is estimated to be 60% for women with deleterious germline *BRCA1* mutations, and 16.5% for women with deleterious germline *BRCA2* mutations, both significantly higher than normal people^6^. Among Chinese ovarian cancer patients, the prevalence of deleterious germline *BRCA1/2* mutations is about 16.7–28.5% in different studies^7–9^. Germline mutations in other HRR genes may also elevate the risk of ovarian cancer, such as *RAD51C*, *RAD51D* and *BRIP1*^10^. The life time risk of ovarian cancer associated with these genes mutations is approximately 6–9%^11,12^. Meanwhile, the deficient in MMR pathway genes predispose carriers to a variety of cancers, including ovarian cancer. The estimated cumulative risks of ovarian cancer for *MLH1* mutation carriers and *MSH2* carriers at age 70 is 20% and 24% respectively^13^. In addition, attention should also be paid to germline mutations in other susceptibility genes associated with hereditary cancer syndromes, such as Li-Fraumeni syndrome (*TP53*), Cowden syndrome (*PTEN*) and Peutz-Jeghers syndrome (*STK11*)^10,14–16^.

Ovarian cancer patients carrying mutations in *BRAC1/2* or other HRR genes may benefit from platinum-based chemotherapy and PARP inhibitors. Patients with detected *BRCA1/2* mutations have a higher response rate to platinum-based chemotherapy and improved median overall survival^17^. Meanwhile, the presence of mutations in other HRR genes (*RAD51C*, *RAD51D*, *BRIP1*, *ATM*, etc.) may also predict favorable prognosis when receiving platinum-based chemotherapy^18^. In addition, patients carrying mutations in *BRAC1/2* or certain HRR genes have improved progression-free survival and overall survival with PARP inhibitors therapy^19–22^. A positive test result may also have important implications for family members, including future generations. Once a *BRCA1* or *BRCA2* germline pathogenic variant has been identified in a family, testing of at-risk relatives can identify those family members who also have the familial pathogenic variant and thus need increased surveillance and specific treatments when a cancer is identified.

Although research in cancer genomics and cancer genetics over the past decade had promoted our understanding of pathogenesis and revolutionized therapies of ovarian cancer, more aspects are yet to be defined. Here, we retrospectively analyzed the germline gene mutations of consecutive ovarian cancer patients in our center (The largest gynecological cancer center in Western China has treated 450–500 newly diagnosed ovarian cancer patients in recent five years) by multi-gene panel detection in BGI genomics, containing HHR, MMR, Fanconi anemia pathway and other DNA damage response genes. The results presented here will expand our knowledge of the impact of germline mutations on heredity and treatment of ovarian cancer.

## Results

### Patient characteristics

A total of 432 patients were tested for germline gene mutations using multi-gene panel. The characteristics of all patients are shown in Table 1 and Supplementary Table S1. The enrolled patients spanned an age range of 20 to 78 years, with a mean age of 52 years (Table 1). *BRCA* deleterious mutations (defined as likely pathogenic/pathogenic mutations) were detected in 126 patients, and among them 41 patients (32.5%) had family history of cancer, including ovarian cancer, breast cancer, colorectal cancer, gastric cancer, etc. In contrast, 71 patients (23.2%) had family history without *BRCA* deleterious mutations (defined as wild type/benign/likely benign mutations). At diagnosis, the median age of all patients was 52 years, with a range of from 20 to 78 years (Table 1 and Supplementary Fig. S1). Among the patients studied, 63% of them were identified as high-grade serous carcinoma (HGSC), while 37% were classified as non-HGSC (Table 1).

**Table 1:** Demographic and clinical characteristics of study population.

	With *BRCA* deleterious mutation (%)	Without *BRCA* deleterious mutation (%)	All
Patients	126 (29.2)	306 (70.8)	432
Family history			
With	41 (32.5)	71 (23.2)	112
Without	85 (67.5)	235(76.8)	320
Age			
Average	52.65	52.92	52.84
Median	51	52	52
Range	30–77	20–78	20–78
Stage			
I & II	29 (23.0)	68 (22.2)	97
III	85 (67.5)	195 (63.7)	280
IV	12 (9.5)	43 (14.1)	55
Pathological type			
HGSC	94 (74.6)	179 (58.5)	273
Non-HGSC	32 (25.4)	127 (41.5)	159

*HGSC* High grade serous carcinoma.

### Overall genetic mutation landscape

Overall, deleterious germline mutations were identified in 346 patients (80.1%), suggesting that deleterious germline mutations in DDR genes are common in ovarian cancer. Moreover, at least one deleterious germline HRR gene mutation was identified in 240 patients (55.6%) and more than one in 80 patients. The most deleterious mutations observed were in *BRCA1* (18.5%), *BRCA2* (12.5%), *ERCC5* (6%), *MLH1* (5.8%), *PALB2* (3.7%) and *ERCC4* (3.7%) 0.155 patients (35.9%) had deleterious mutations in germline HRR genes other than *BRCA1/2*. Germline MMR genes deleterious mutations were identified in some patients, including *MLH1* mutations in 25 patients (5.8%), *MSH6* mutations in 6 patients (1.4%), *MSH2* mutations in 4 patients (0.9%) and *PMS2* mutations in 3 patients (0.7%). Notably, HGSC demonstrated a significantly higher incidence of pathogenic *BRCA1* mutations (P-value < 0.005), while non-HGSC was characterized by a higher frequency of pathogenic *NCOA4* mutations (P-value < 0.006).

**Figure 1:**
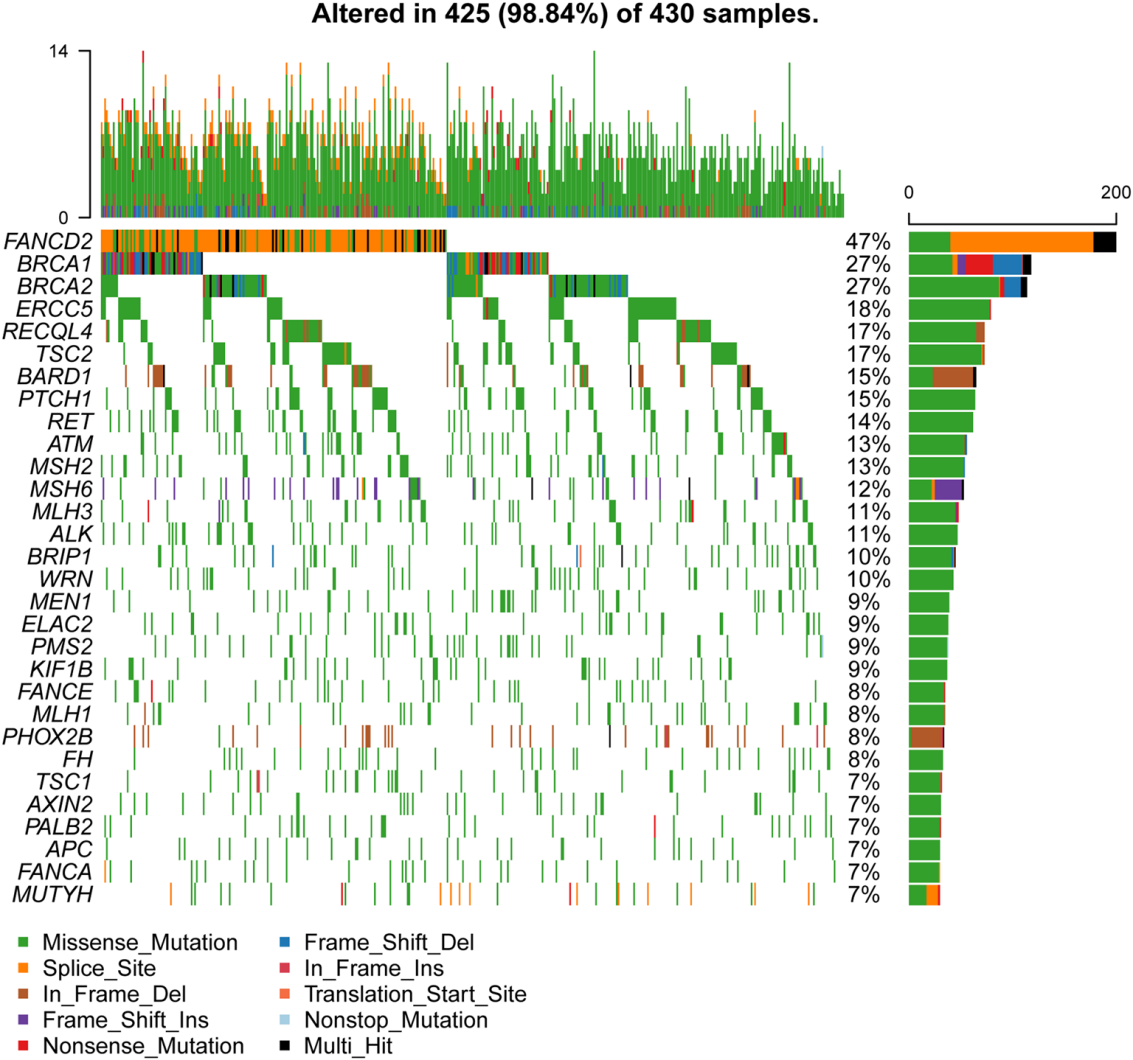
Mutational landscape of our study cohort.

When considering all the mutations, not only deleterious, *FANCD2* (47%), *BRCA1* (27%), *BRCA2* (27%), *ERCC5* (18%) and *RECQL4* (17%) were the most frequently mutated genes (Fig. 1). The frequency of missense mutation was higher than them of other mutation types, except for a few genes. *FANCD2* had high frequency of splice site mutation, and *TSC2* and *PHOX2B* had high frequency of in frame deletion. High frequent mutations were enriched in DNA damage repair signal pathway, such as Fanconi anemia, HRR, nucleotide excision repair (NER), MMR and cell cycle pathway (Supplementary Table S2). And 88.2% patients were implicated by at least one Fanconi anemia pathway gene mutation.

Using the pair-wise Fisher's Exact test to explore the co-occurrence and mutually exclusive interaction of mutations between genes, it was found that the mutations in *ALK* and *BARD1* were significantly exclusive each other in the same patient, as well as *KIF1B* and *MSH6*; whereas, the mutations in the *FH* and *MLH1*, along with *BARD1* and *PMS2*, were significantly co-occurring in the same patient (Supplementary Fig. S2).

### *BRCA1/2* mutation sites and novel mutations

**Figure 2:**
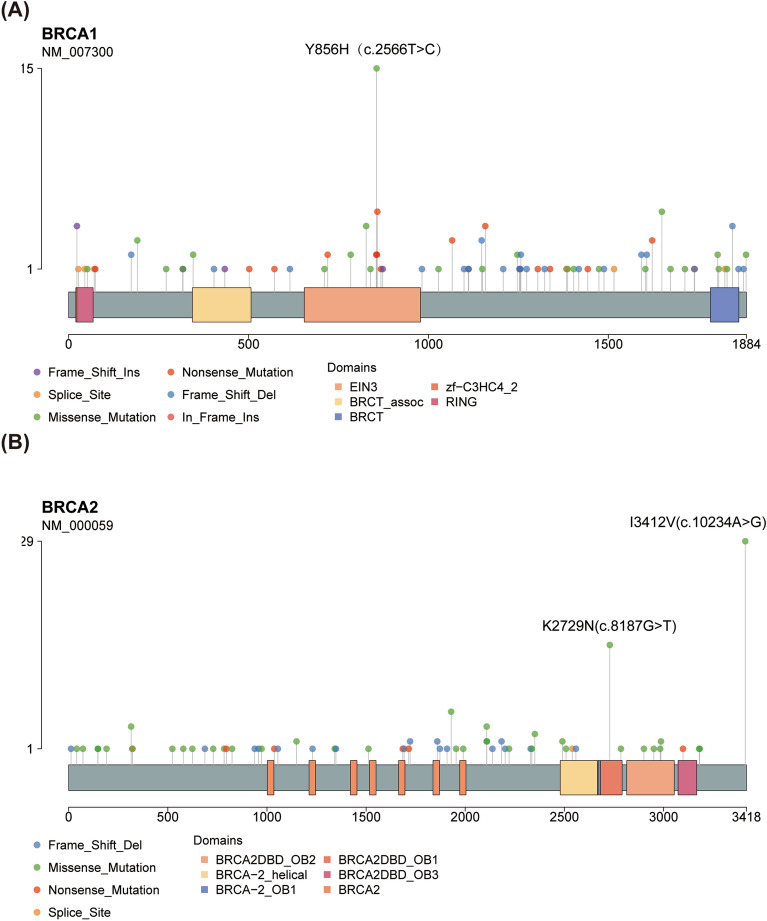
*BRCA1/2* founder mutations in our study cohort. (**A**) *BRCA1* deleterious mutations. (**B**) *BRCA2* deleterious mutations.

**Figure 3:**
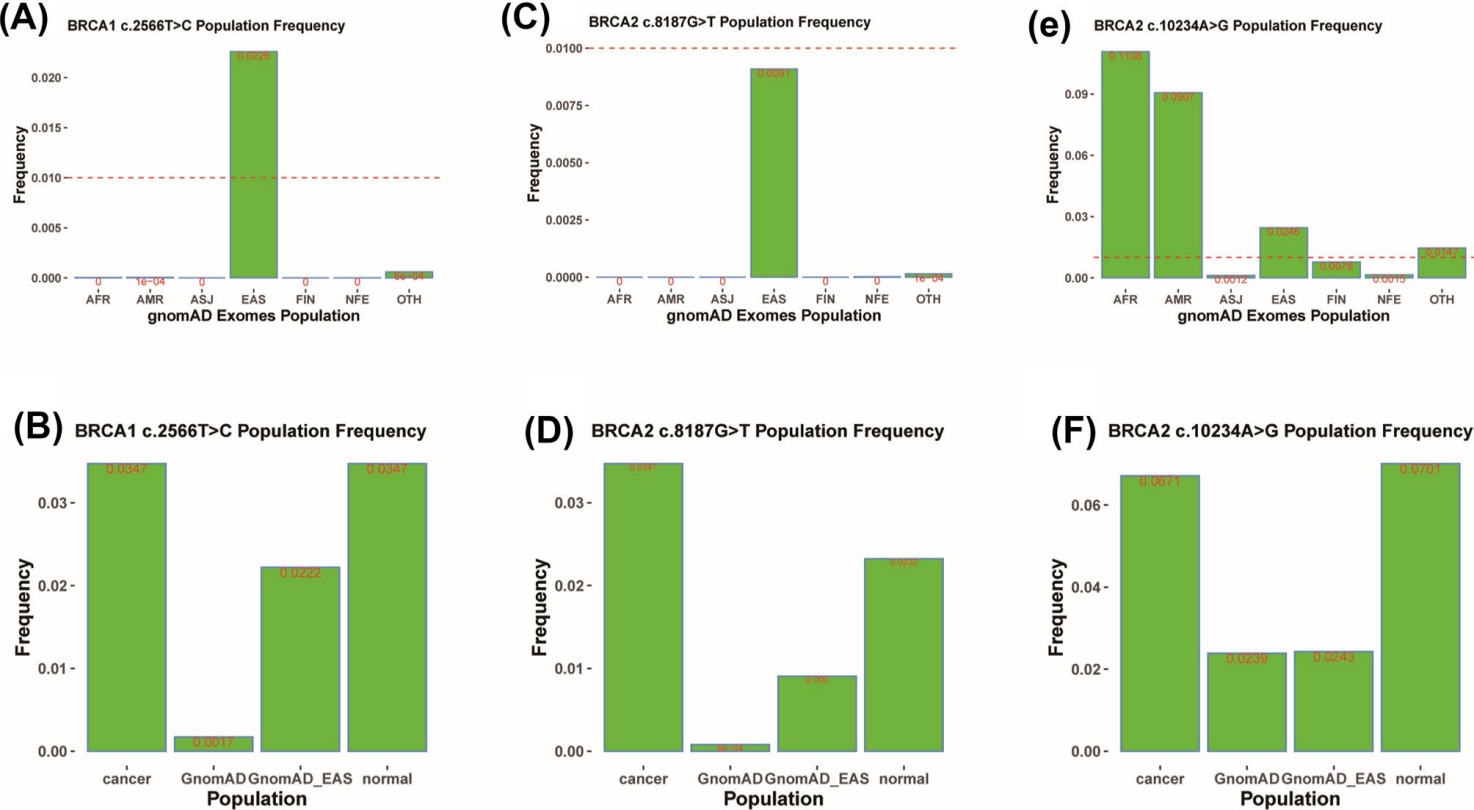
*BRCA1/2* founder mutations in in different population. (**A**) Frequency of *BRCA1* c.2566 T > C in different population. (**B**) Frequency of *BRCA1* c.2566 T > C in Chinese ovarian cancer patients and normal people. (**C**) Frequency of *BRCA2* c.8187G > T in different population. (**D**) Frequency of *BRCA2* c.8187G > T in Chinese ovarian cancer patients and normal people. (**E**) Frequency of *BRCA2* c.10234A > G in different population. (**F**) Frequency of *BRCA2* c.10234A > G in Chinese ovarian cancer patients and normal people. *AFR* African/African American, *AMR* Latino/Admixed American, *ASJ* Ashkenazi Jewish, *EAS* East Asian, *FIN* European (Finnish), *NFE* European (non-Finnish), *OTH* Other.

After comprehensively analyzing the mutation sites of *BRCA1* and *BRCA2*, we found that the incidence of *BRCA1* c.2566 T > C was significantly higher than that of other mutations, converting tyrosine at 856 site to histidine (Fig. 2A). And when analyzed in population mutation frequency database, *BRCA1* c.2566 T > C was significantly enriched in Asian population (Fig. 3A). In the Chinese population, *BRCA1* c.2566 T > C was more frequent in ovarian cancer patients than healthy controls (Fig. 3B). Meanwhile, *BRCA2* c.8187G > T and c.10234A > G had remarkably higher frequency than other mutated sites, converting lysine at 2729 site to asparagine and isoleucine at 3412 site to valine (Fig. 2B). The frequency of *BRCA2* c.8187G > T was specifically higher in Asian population than other mutated sites and moderately higher in Chinese ovarian cancer patients than healthy people (Fig. 3C,D). *BRCA2* c.10234A > G was a high-frequency mutation in African and American populations (Fig. 3E). The frequency of *BRCA2* c.10234A > G observed in Chinese ovarian cancer patients was 1.64 times higher than that of in normal people (Fig. 3F).

In our study cohort, we confirmed three novel germline mutations in *BRCA1/2* in ten patients that are not reported in public databases and literature (Supplementary Table S3). Two of ten patients had a family history of cancers. *BRCA1* p.M1649T, p.K1622* and *BRCA2* p.L2987P was identified in five patients, three patients and two patients, respectively. *BRCA1* p.M1649T and *BRCA2* p.L2987P were missense mutations, while *BRCA1* p.K1622* was a nonsense mutation. We predicted whether these novel germline mutations were deleterious using SIFT^23^ and PolyPhen2^24^. *BRCA1* p.M1649T was not a deleterious mutation, but *BRCA2* p.L2987P was. However, the software-predicted pathogenicity of *BRCA1* p.K1622* was unknown. Considering *BRCA1* p.K1622*truncated the protein, we think it is probably a deleterious mutation.

### High frequency* FANCD2* mutation

*F*ANCD2 was the most mutated gene in our data, with 73.3% of patients carried four adjacent cis mutations c.1275C > T, c.1278 + 1delG, c.1278 + 3_1278 + 5delAAG and c.1278 + 15C > T, and all of these carries were heterozygous. *FANCD2* c.1275C > T was a silent mutation didn’t alter *FANCD2* protein sequence, and the other three mutations affected intron 16 of *FANCD2*. In ClinVar database, *FANCD2* c.1275C > T, c.1278 + 3_1278 + 5delAAG and c.1278 + 15C > T, were labeled as benign mutations, while c.1278 + 1delG had no clinically reported significance. As GT dinucleotides were the most common 5’ splice site of intron, deletion of the first guanine would lead to splicing at wrong site. Thus, c.1278 + 1delG may dramatically impact the final transcript and function of *FANCD2*. Although these adjacent mutations alone were detected and reported in population database, cis mutation type of the four mutations were not reported and they may have additive effect to the function of *FANCD2*. We combined them as *FANCD2* span mutation c.1275_1278 + 15delCTTAGTAAGTGTCAGAGACinsTTTATTGTCAGAGAT, which spanned both the exon and intron. According to the ACMG guidelines for the interpretation of sequence variants, this *FANCD2* span mutation was likely pathogenic. We applied fit Chi-square calculation to evaluate the mutation with ovarian cancer risk and found the mutation of *FANCD2* that significantly fails to conform Hardy–Weinberg equilibrium (P-value 2.76e − 06).

### Mutation characteristic of subgroups with different ages at diagnosis

**Figure 4:**
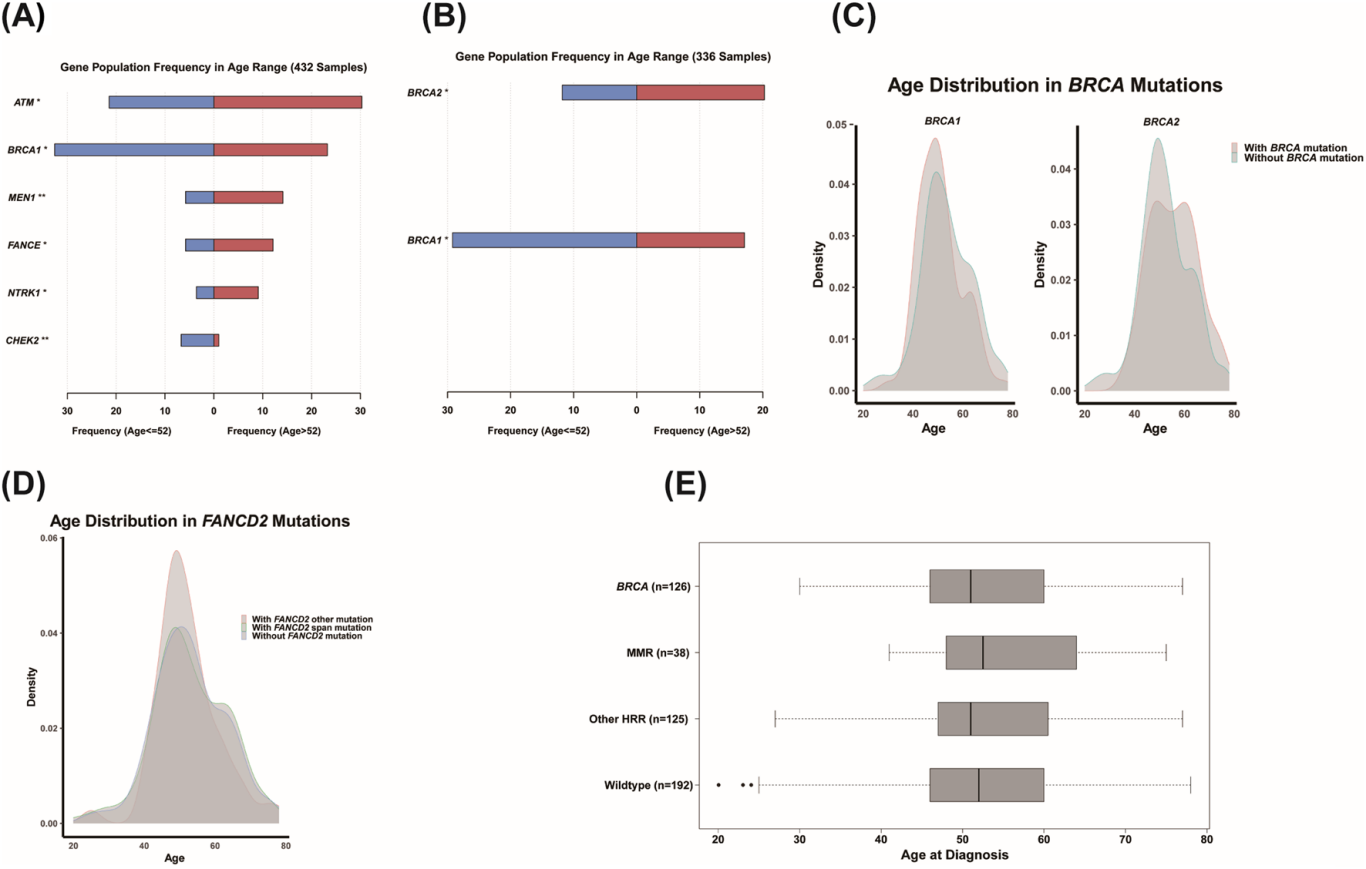
Mutation characteristic different age groups. (**A**) Significant mutant genes in ovarian cancer patients with different ages (separated by 52 years old). (**B**) Frequency of *BRCA1/2* deleterious mutations in ovarian cancer patients with different ages (separated by 52 years old). (**C**) Age distribution of patients detected *BRCA1/2* deleterious mutations. (**D**) Age distribution of patients detected *FANCD2* mutations. (**E**) Age distribution of patients detected DNA damage response genes. *Indicates that the P-value is less than 0.05, **Indicates that the P-value is less than 0.01.

Furthermore, we investigated the association between germline mutations and age at diagnosis. In all patients, *BRCA1* and *CHEK2* mutations were more common in younger patients, whereas *ATM*, *MEN1*, *FANCE* and *NTRK1* were mutated more in older patients (Fig. 4A). Germline mutations in *BRCA1* and *CHEK2* may lead to homologous recombination repair deficiency and cell cycle disorder, eliciting cancer at an early age. Then, we focused on deleterious mutations of *BRCA1* and *BRCA2*, the most crucial genes increasing the risk of ovarian and breast cancer. Among the 336 patients carrying deleterious mutations, *BRCA1* was with higher frequency in younger patients, while *BRCA2* was moderately higher in older patients (Fig. 4B,C). We also observed that patients with *FANCD2* span mutation were slightly younger than those without *FANCD2* mutations, although not significantly, suggesting that *FANCD2* span mutation may be associated with early onset of ovarian cancer (Fig. 4D). When analysis based on different signal pathways, patients with HRR deleterious mutations were younger than them with MMR deleterious mutations, meanwhile younger than them without HRR and MMR deleterious mutations (also named “wild type”) (Fig. 4E).

### Mutation characteristic of subgroups with or without a family history

**Figure 5:**
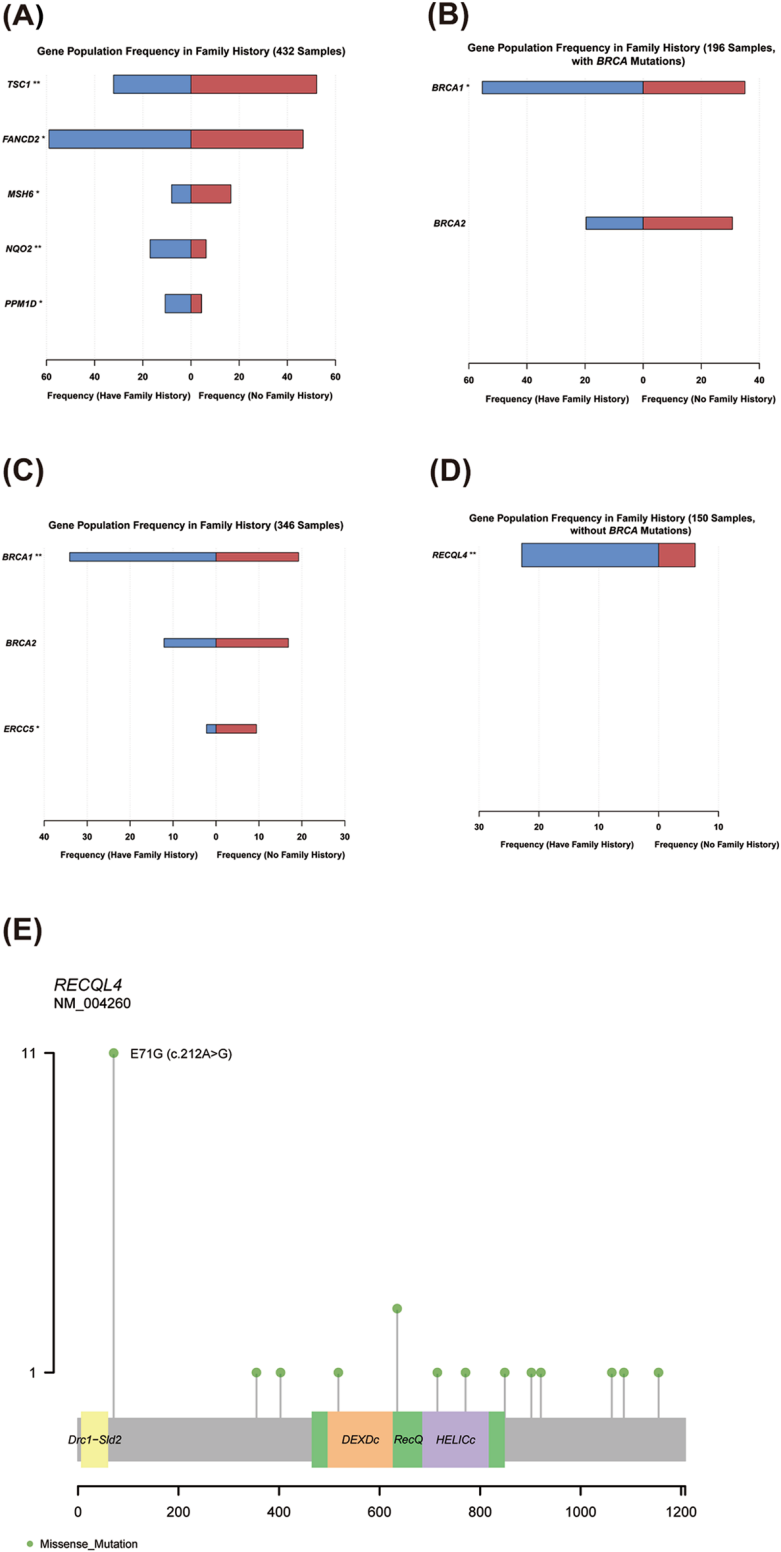
Different gene mutations in patients with or without family history. (**A**) Significant mutated genes. (**B**) Significant mutated genes in patients carrying deleterious mutations. (**C**) Significant mutated genes in patients carrying *BRCA1/2* deleterious mutations. (**D**) Significant mutated genes in patients not detected *BRCA1/2* deleterious mutations. (**E**) *RECQL4* deleterious mutations. *Indicates that the P-value is less than 0.05, **Indicates that the P-value is less than 0.01.

There are 112 patients with a family history of cancer. In all patients, *FANCD2*, *NQO2* and *PRM1D* were commonly mutated in patients with a family history, while *TSC1 *and *MSH6* mutations were occurred frequently in patients without a family history (Fig. 5A). When we focused on patients with deleterious mutations, *BRCA1* was significantly associated with family history, and *BRCA2* was slightly more common in patients without a family history but not significantly (Fig. 5B,C). Moreover, *ERCC5*, an endonuclease participating the excision repair pathway, was observed more frequently in patients without a family history (Fig. 5B).

In patients without *BRCA* deleterious mutations, *RECQL4* deleterious mutations were significantly accompanied with family history (Fig. 5D). In our cohort, *RECQL4* deleterious mutations were observed in 5.6% of patients, of which 45.8% was c.212A > G (p.E71G) (Fig. 5E). Thus, *RECQL4* deleterious mutations, especially the mutation c.212A > G may predispose carriers to ovarian cancer.

## Discussion

The prevalence and spectrum of germline mutations in ovarian cancer vary across population and studies. *BRCA1* and *BRCA2* are the most studied ovarian cancer susceptibility genes, and are associated with hereditary breast and ovarian cancer syndrome. The reported germline *BRCA1/2* mutation prevalence in ovarian cancer is ranged from 5.6% to 29.3%, with the highest frequency in Ashkenazi Jews^25^. And except for Poland, the frequency of germline *BRCA1/2* mutation is relatively low in Northern European countries (Finland, Sweden, Denmark and Iceland), which are less than 10%^25^. The frequency of germline *BRCA1/2* mutation in Chinese ovarian cancer patients is 16.7–28.5%, similar to that in other Eastern Asia countries (14.6% in Japanese and 19.5% in Korean)^7–9,25^. Lynch syndrome is another hereditary cancer syndrome associated with gynecologic cancers, which is owing to the deleterious germline mutations in MMR genes, and underlying approximately 5% of endometrial cancers and 1% of ovarian cancers^26,27^. In our cohort, at least one deleterious mutation was detected in 346 (80.1%) patients using multi-gene panel. The most observed germline deleterious mutation was *BRCA1*, with a frequency of 18.5%, which is similar to 13.1–20.8% in other studies^7–9^. However, the frequency of germline *BRCA2* deleterious mutation was 12.5%, higher than 3.9–7.6% in other studies, which is probably due to the different geographical distribution of patients^7–9^. The patients in our cohort were mainly from Southwest China, while in other studies they were primarily from Eastern China. *BRCA1* pathogenic mutations have been reported to be significantly more prevalent in HGSC^7,9^, which is consistent with our analysis. Meanwhile, our research revealed a significantly higher prevalence of pathogenic *NCOA4* mutations in non-HGSC. The relationship between *NCOA4* mutations and ovarian cancer has not yet been thoroughly investigated. Previous study has reported endometrial cancer populations harboring *NCOA4* mutations are associated with a more favorable prognosis compared to wild-type^28^. We also observed higher germline deleterious MMR gene mutations in our study, indicating that lynch syndrome may be more frequently associated with ovarian cancer patients of Southwest China. Thus, we should be concerned more about their personal and family history of lynch syndrome associated cancers, such as endometrial cancer and colorectal cancer.

When analyzing specific mutation sites in *BRCA1* and *BRCA2*, we identified high frequency mutation sites, *BRCA1* c.2566 T > C, *BRCA2* c.8187G > T and *BRCA2* c.10234A > G. They were missense mutations and were not currently considered as deleterious mutations. *BRCA1* c.2566 T > C converted tyrosine to histidine. Tyrosine was a non-essential amino acid and required energy to synthesize, while histidine was an essential amino acid mainly acquired from food. Therefore, the *BRCA1* mutation, c.2566 T > C, reduced the energy consumption of cancer cells and promoted to proliferation. We identified three novel *BRCA1/2* mutations in ten patients, each of which was observed in at least two unrelated patients. Taking into account software prediction, effects on protein function and family history, *BRCA1* p.K1622* and *BRCA2* p.L2987P may be germline deleterious mutations. Further detailed studies are needed to clarify the impact of these mutations on carriers and ovarian cancer patients.

In addition to the known ovarian cancer high-risk genes, we found that germline mutations in *FANCD2* and *RECQL4* may be associated with hereditary and early onset of ovarian cancer. *FANCD2* is a key protein in Fanconi anemia signal pathway and plays an important role in many aspects of cell life, especially in DNA damage response^29^. Upon activation, *FANCD2* is monoubiquitinated and forms heterodimer with *FANCI*, leading to signal amplification and downstream repair protein recruitment^30^. We identified a germline likely pathogenic *FANCD2* span mutation, comprised of our adjacent *cis* mutations. 47% of patients carried *FANCD2* mutations, of which 73.3% had the *FANCD2* span mutation. *FANCD2* span mutation located on a 23 nucleotides fragment across exon and intron, may affect protein function together. And the *FANCD2* span mutation may greatly influence protein function due to wrong splice site. Our data also indicated that *FANCD2* mutations, especially the *FANCD2* span mutation, were related to family history and early onset of ovarian cancer. Moreover, Fanconi anemia signaling pathway is involved in the repair of inter strand crosslink, which may be induced by exposure to environmental mutagens or commonly used chemotherapeutic agents of cancers, such as platinum and furocoumarins^31,32^. Therefore, *FANCD2* mutations potentially affect patients’ prognosis. Over expression of *FANCD2* was reported as a strong negative prognostic factor in ovarian cancer, particularly in patients treated with taxane-platinum^33^.

*RECQL4* belongs to RecQ helicase family, which plays important roles in multiple cell life processes, including DNA replication, transcription, DNA repair, and telomere maintenance^34,35^. *RECQL4* mutations are associated with three rare autosomal-recessive syndromes, namely Rothmund–Thomson syndrome, RAPADILINO and Baller–Gerold syndrome^35,36^. All these three syndromes are correlated to cancer, particularly Rothmund–Thomson syndrome patients with *RECQL4* mutations shown an increased risk of osteosarcoma^35,36^. For Rothmund–Thomson syndrome patients with *RECQL4* mutations, the leading cause of death mutations is cancer, whereas in cancer-free patients the life expectancy is normal^34^. Recent studies also reported that germline deleterious mutations in *RECQL4* are associated with predisposition to breast cancer and prostate cancer^37–41^. Besides, *RECQL4* plays a role in HRR of DNA double strand breaks^42^. Abnormal *RECQL4* may affect the prognosis of patients treated with platinum or PARP inhibitors^43,44^. In our study, germline deleterious mutations in *RECQL4* were significantly associated with a family history in patients without *BRCA1/2* mutations, suggesting that it may be also related to ovarian cancer susceptibility. In our cohort, germline deleterious mutations in *RECQL4* were detected in 24 patients (5.6%), nearly half of which were c.212A > G. *RECQL4* c.212A > G mutation was not a known mutation in the three autosomal-recessive syndromes, and its roles in ovarian carcinogenesis requires further investigation^45,46^.

In conclusion, the results presented here showed the germline DDR gene mutation spectrum of ovarian cancer patients in Southwest China, which is genetically different from the rest of China. We also identified novel *BRCA* mutations worthy of our further attention. Finally, we found that germline deleterious mutations in *FANCD2* and *RECQL4* were likely susceptibility genes for ovarian cancer, and we should be more cautious to these two genes.

The observed regional and population disparities in the prevalence of deleterious germline mutations, particularly in *BRCA2* and mismatch repair genes, emphasize the significance of regional genetic variations in ovarian cancer (OC) susceptibility. This study highlights the necessity for comprehensive germline mutation testing in DNA damage response (DDR) genes for OC patients, enhancing patient management and genetic counseling. It advocates for healthcare providers to extend their genetic assessments to a broader spectrum of DDR genes, not limited to *BRCA1/2*. Future research should explore the functional implications of newly identified mutations and their influence on OC risk. Moreover, expansive studies across diverse populations are essential to validate the observed regional and population variances in DDR gene mutations, potentially guiding personalized treatments and better outcomes for OC patients.

## Methods

### Participants

We retrospectively and unselectively enrolled a cohort of patients diagnosed with advanced epithelial ovarian cancer from October 2016 to October 2020. All enrolled patients were required to meet the following criteria: (1) A pathologically confirmed diagnosis via frozen section or paraffin section or ascites cells by two expert pathologists, meeting the pathological diagnosis criteria for EOC (including serous carcinoma, endometrioid carcinoma, clear cell carcinoma, mucinous carcinoma, undifferentiated carcinoma, and carcinosarcoma); (2) Advanced radiographic imaging (CT, MRI, PET) results met primary EOC characteristics; (3) Sufficient medical history and demographic data; (4) Not undergoing comprehensive staging surgery/tumor cell debulking surgery, chemotherapy, radiotherapy, targeted therapy. Exclusion criteria included: (1) Concurrent other malignancies; (2) Incomplete clinical and pathological data; (3) Other pathological types of ovarian cancer (or non-EOC). Clinical information (age, family history, and personal history), pathology information (pathological diagnosis, tumor FIGO stage), were captured and summarized. All Eligible patients had signed informed consent for gene testing. In total, peripheral blood samples from 432 patients were collected for evaluating the specific germline alterations. Based on the current sample size, a sample power of 1 was confirmed by the PWR package in R. Blood samples were collected before any surgical or chemotherapy treatment. Each patient used an EDTA anticoagulant tube to collect 5 ml of peripheral blood, mixed it by inversion, and sent it to the testing laboratory within 5 days under conditions of 2 to 8℃. This study was approved by the Medical Ethics Committee of West China Second University Hospital, Sichuan University (Ethical Lot Number 20200076). The research was carried out according to guidelines and regulations of the ethic committee.

### Next-generation sequencing

Blood samples were collected at West China Second University Hospital and sent to BGI Shenzhen Clinical Diagnostic Laboratory (the contract central testing laboratory for this study), where DNA extraction, targeted DNA sequencing, variant calling, and interpretation were performed. Briefly, genomic DNA (gDNA) was extracted from participants’ peripheral blood using the Qiagen Blood Midi Kit (Qiagen, Hilden, Germany) according to the manufacturer’s standard protocol. DNA concentration and quality were assessed by Qubit (Life Technologies, Carlsbad, USA) and agarose gel electrophoresis. The gDNA (250 ng) was randomly fragmented by the Covaris LE220 sonicator (Covaris, Woburn, USA) to generate gDNA fragments with a peak of 250 bp and then subjected to three enzymatic steps: end-repair, A-tailing, and sequencer (MGI, Shenzhen, China) adapter ligation. DNA libraries were purified with Agencourt Ampure XP beads (Beckman-Coulter, Indiana, USA), and PCR was carried out to form a pre-PCR library or pre-hybridization library, during which a unique 8 bp barcode was added to label each sample. Five to ten pre-PCR libraries were pooled equally and hybridized to a custom hereditary cancer panel (BGI, Shenzhen, China). After purification, the enriched DNA was specifically captured and amplified by PCR to obtain a post-PCR library. The post-PCR library were subjected to single-strand separation, circularization and rolling circle replication to generate DNA nano balls (DNB) and sequencing was performed with 2 × 101 bp paired-end reads on a BGISEQ-500 or MGISEQ-2000 platform (MGI, Shenzhen, China) following the manufacturer’s protocols. SNVs and INDELs in all coding exons and intron–exon boundaries (± 20 base pairs) of detected genes were identified from NGS data.

### Sequencing data analysis

Raw fastq data generated by the sequencer was first filtered by SOAPnuke 1.5.0 to exclude low quality reads^47^. The clean reads were then aligned to the reference human genome (UCSC hg19) using the Burrows-Wheeler Aligner (BWA 0.7.12) MEM algorithm^48^. PCR deduplication was performed using Picard 1.87. The average depth was over 100X and the coverage at 30X exceeded 95% for each sample. Single-nucleotide variants (SNVs), small insertions and deletions (INDELs) were detected by Genome Analysis Toolkit (GATK 4.0.8.1) HaplotypeCaller^49^. SNVs and INDELs in all coding exons and intron–exon boundaries (± 20 base pairs) of detected genes were identified from NGS data. All of above variants were further filtered by quality depth, strand bias, mapping quality and read position. Finally, each variant was annotated by Bcfanno for gene location and predicted function in Human Genome Variation Society (HGVS) nomenclature and was ready for interpretation.

### Data interpretation

Variants were classified into the following five categories according to the American College of Medical Genetics (ACMG) recommendations: class 1, benign; class 2, likely benign; class 3, variant of unknown significance (VUS); class 4, likely pathogenic; and class 5, pathogenic^50^. Likely pathogenic and pathogenic variants will be verified by Sanger sequencing. Individuals with likely pathogenic or pathogenic variants were defined as having deleterious variants.

### Statistical analysis

All statistical analysis and plots were conducted using R version 3.6.3. Pearson's χ^2^-test was used as a statistical significance test in the analysis of categorical variables. We performed Student’s t-test to compare continuous variables between two groups, such as age at diagnosis. Patients with specific missing clinical data were excluded from the relevant analysis of specific clinical characteristic. Each reported P value was two-sided and P < 0.05 was considered to be statistically significant.

### Ethics statement

The studies involving human participants were reviewed and approved by Medical Ethics Committee of West China Second University Hospital (Ethical Lot Number 20200076). The patients/participants provided their written informed consent to participate in this study.

### Supplementary Information

Supplementary Information.

Supplementary Table S1.

## Data Availability

The data that support the findings of our study are available in the Supplementary material. The sequencing data generated and analysed during the current study are available in the ClinVar, accession numbers SCV003843260—SCV003843860.
