# Supplementary material for: Characteristics of germline DNA damage response gene mutations in ovarian cancer in Southwest China
Source: Sci Rep. 2024 Mar 20;14:6702. doi: 10.1038/s41598-024-52707-y (PMC10954728; doi:10.1038/s41598-024-52707-y)
Supplement: Supplementary file 1 — Supplementary Information. [file 41598_2024_52707_MOESM1_ESM.pdf]

Supporting Information:

Characteristics of germline DNA damage response gene mutations in ovarian cancer in Southwest China

Kaiyu Fu[1,2], Qingli Li[1,2], Jie Wang[3,4], Mengpei Zhang[1,2], Xinyu Yan[3], Kemin Li[1,2], Liang Song[1,2], Lan Zhong[1,2], Yu Ma[1,2], Jinghong Chen[1,2], Jing Zeng[1,2], Danqing Wang[1,2], Di Shao[3], Shida Zhu[3], Rutie Yin[1,2]

Kaiyu Fu, Qingli Li and Jie Wang contributed equally to this article

[1] Department of Obstetrics and Gynecology, West China Second University Hospital, Sichuan University, Chengdu, Sichuan, China

[2] Laboratory of Molecular Epidemiology of Birth Defects, West China Second University Hospital, Sichuan University, Chengdu, Sichuan, China

[3] BGI Genomics, BGI-Shenzhen, Shenzhen, China

[4] College of Life Sciences, University of Chinese Academy of Sciences, Beijing, China

Corresponding author:

Rutie Yin, Department of Obstetrics and Gynecology, West China Second University Hospital, Sichuan University, Chengdu, Sichuan, 610041, China. Email: yrtt2013@163.com

Shida Zhu, BGI Genomics, BGI-Shenzhen, Shenzhen 518083, China. Email: zhushida@genomics.cn

Table S1: Mutation and clinical information

Table S2: Signal pathways of gene enrichment

Pathways	Number of genes	P value
**Fanconi anemia**	16	< 0.001
**Cell cycle**	9	< 0.001
**HRR**	8	< 0.001
**NER**	7	< 0.001
**MMR**	5	< 0.001

Table S3: BRCA1 and BRCA2 novel mutations

No. of patients	Gene	Mutation type	Mutation site	Frequency	Age	Deleterious	Family history
**No. 1**	BRCA1	missense	p.M1649T	0.487464	56	No	No
**No. 2**	BRCA1	missense	p.M1649T	0.491561	50	No	No
**No. 3**	BRCA1	missense	p.M1649T	0.502008	45	No	No
**No. 4**	BRCA1	missense	p.M1649T	0.492	51	No	No
**No. 5**	BRCA1	missense	p.M1649T	0.46	44	No	No
**No. 6**	BRCA1	nonsense	p.K1622*	0.510164	44	Unknown	Yes
**No. 7**	BRCA1	nonsense	p.K1622*	0.473684	48	Unknown	No
**No. 8**	BRCA1	nonsense	p.K1622*	0.492	51	Unknown	No
**No. 9**	BRCA2	missense	p.L2987P	0.429719	Unknown	Yes	No
**No. 10**	BRCA2	missense	p.L2987P	0.424893	64	Yes	Yes

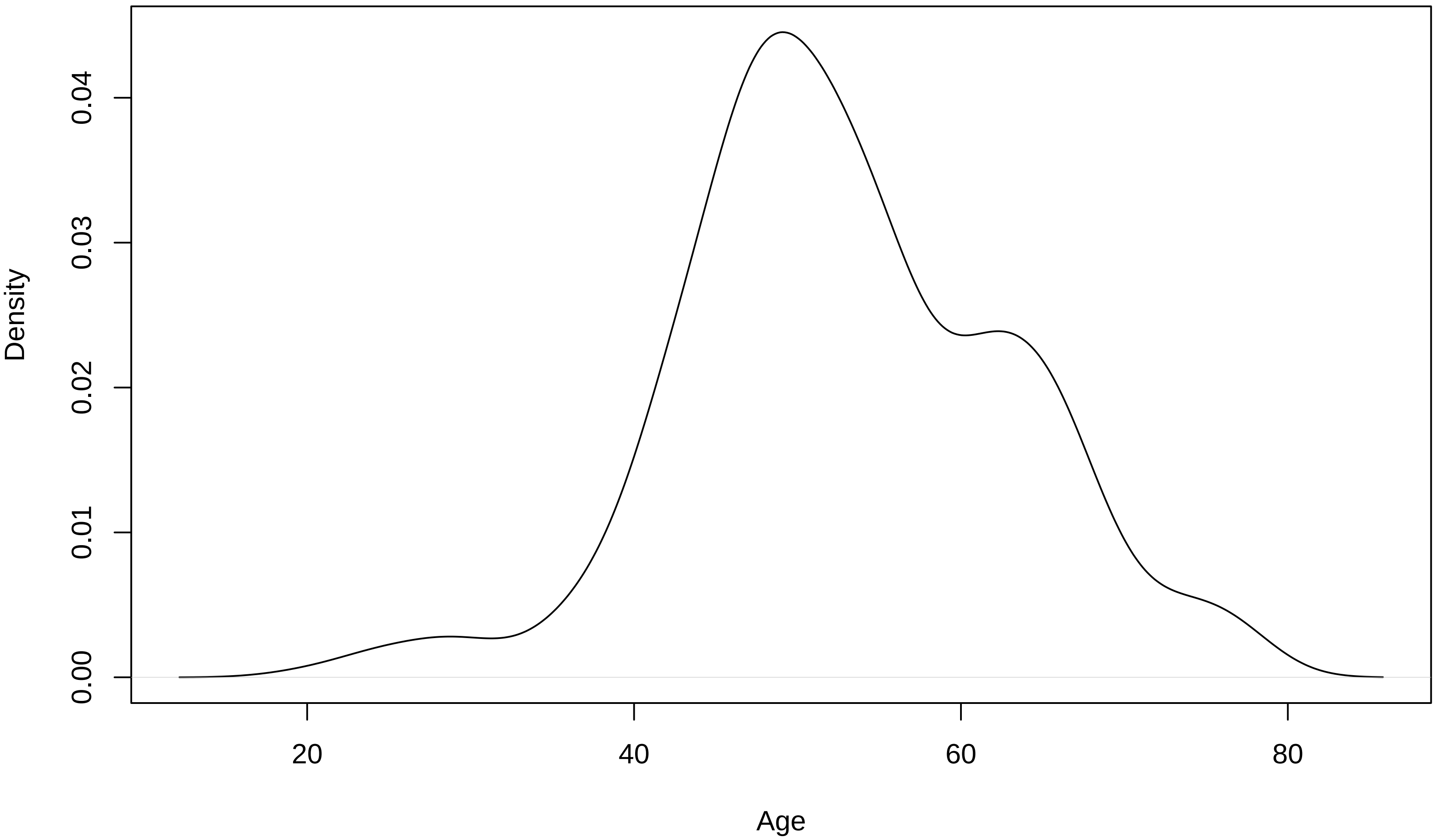

Figure S1: **Age distribution of the sample**

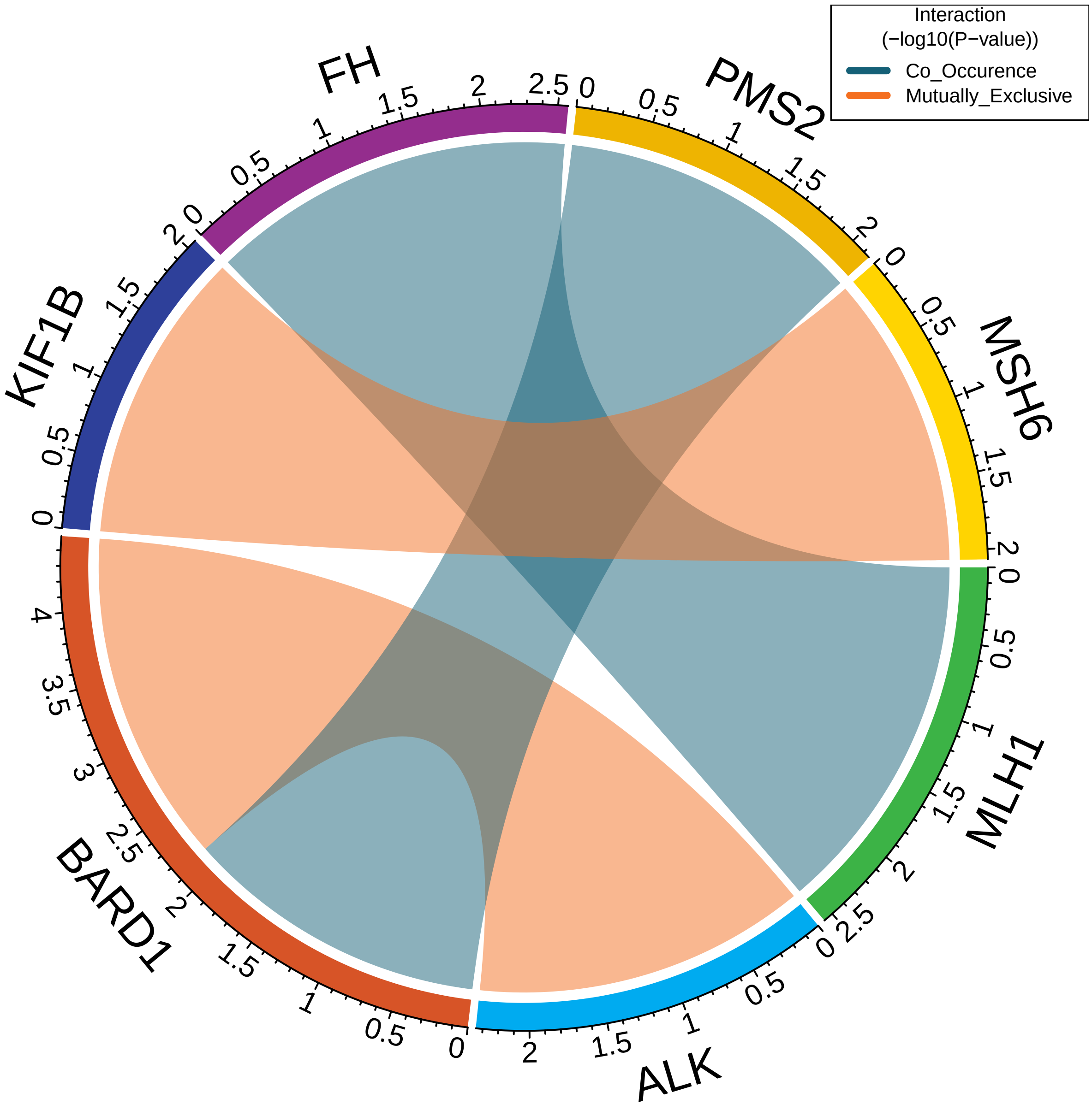

Figure S2: **Mutation co-occurring and exclusive genes in ovarian cancer**
